# Secondary Sclerosing Cholangitis (SSC): An Underrecognized Complication of Severe COVID-19 Pneumonitis

**DOI:** 10.7759/cureus.70003

**Published:** 2024-09-23

**Authors:** Mariya Pogorelova, Victoria Kusztos

**Affiliations:** 1 Department of Medicine, Mayo Clinic, Rochester, USA

**Keywords:** covid-19 liver injury, covid-ssc, elevated alkaline phosphatase, secondary sclerosing cholangitis in critically ill patients(ssc-cip), secondary sclerosing cholangitis (ssc)

## Abstract

COVID-19-secondary sclerosing cholangitis (COVID-SSC) is a distinct subset of secondary sclerosing cholangitis in critically ill patients (SSC-CIP) that presents after COVID-19 infection with alkaline phosphatase predominant elevation of liver enzymes. COVID-SSC typically presents within three months of COVID-19 diagnosis and most commonly occurs following severe COVID-19 infection. COVID-SSC can have different clinical degrees of severity, ranging from clinically latent, as shown in this case report, to severely symptomatic, requiring a liver transplant or leading to patient death.

We present a case of COVID-SSC that presented in an asymptomatic patient months after severe COVID pneumonitis requiring prolonged intubation who was initially misdiagnosed with autoimmune hepatitis and found to have early cirrhosis at the time of diagnosis. The case presented was initially clinically silent and overlooked for months. In the aftermath of severe COVID-19 infection, COVID-SSC should be included in the differential diagnosis of unclear cholestasis, and general practitioners should have a high index of suspicion when encountering disproportionate elevation of alkaline phosphatase in patients with a history of COVID-19, in particular, those requiring intensive care unit (ICU) level cares.

## Introduction

Secondary sclerosing cholangitis in critically ill patients (SSC-CIP) is an increasingly recognized etiology of secondary sclerosing cholangitis (SSC) that develops in patients without prior biliary disease following intensive care treatment. It is unique from other causes of SSC as there is no evidence of pathology causing biliary obstruction, and it is characterized by cholestasis, bile duct necrosi­­s, and progression to biliary cirrhosis and liver failure [1]. Initial teaching was that SSC-CIP typically occurs within months of prolonged ICU care, most commonly in patients treated for infection, burns, and trauma. More recently published studies support that SSC is not a late complication of an intensive care course but acute ischemia of the intrahepatic bile ducts that can be noticed during first few days of ICU admission. Factors common to ICU care that put patients at risk for biliary ischemia include hypotension and hemodynamic instability, mechanical ventilation, and microcirculatory disruption such as increased blood viscosity, red blood cell (RBC) transfusions, and hypercoagulable states. Patients with severe COVID-19 are exposed to many of these risk factors. Cholangiocytes are susceptible to SARS-Cov-2, suggesting a mechanism direct viral damage. Initial symptoms of cholestasis can progress to biliary cirrhosis and liver failure and may ultimately require liver transplantation. COVID-19-secondary sclerosing cholangitis (COVID-SSC) has been described as a confluence of SSC-CIP and direct hepatic injury from COVID-19, given their similar clinical phenotype and disease course [2]. COVID-SCC typically develops around three months after COVID-19 diagnosis and portends a poor prognosis as transplant free survival is low [3]. Here, we present a mild case of COVID-SCC that presented one year after severe COVID-19 and was initially misdiagnosed as autoimmune hepatitis.

## Case presentation

Case presentation

A 43-year-old male with a history of hypertension presented for evaluation of alkaline phosphatase predominant elevation of liver enzymes. He was generally healthy until he developed severe COVID-19 pneumonitis, requiring intubation and a seven-week ICU stay. Prior to this hospitalization, his liver enzymes were within normal limits, and he drank approximately two alcoholic beverages per day. During hospitalization, his liver enzymes increased and then normalized by the time of discharge. While recovering from COVID-19, he suffered from peripheral nerve damage, vocal cord paralysis, and long-COVID symptoms, including fatigue and brain fog. Seven months after discharge, laboratory values were incidentally found to reveal the following (reference ranges provided parenthetically): alkaline phosphatase 795 U/L (40-129 U/L), alanine transaminase (ALT) 274 U/L (7-55 U/L), aspartate aminotransferase (AST) 133 U/L (7-55 U/L), and total bilirubin 1.7 mg/dL (<1.2 mg/dL), (Table 1). The patient was asymptomatic without any overt signs of hepatic decompensation. These abnormalities persisted with alkaline phosphatase 637 U/L (40-129 U/L), ALT 147 U/L (7-55 U/L), AST 116 U/L (7-55 U/L), and total bilirubin of 2 mg/dL (<1.2 mg/dL), five months later (Table 1). A magnetic resonance cholangiogram performed 11 months after discharge revealed early cirrhosis with sequelae of portal hypertension, including splenomegaly and mild hepatic geographic steatosis. Two months later, he presented to an emergency room with right upper quadrant abdominal pain, and was diagnosed with acute cholecystitis and choledocholithiasis, which was treated with cholecystectomy, endoscopic retrograde cholangiopancreatography (ERCP), and biliary sphincterotomy with stent placement. Liver enzymes were again elevated at that time with alkaline phosphatase 278 U/L (40-129 U/L), ALT 133 U/L (40-129 U/L), AST 77 U/L (7-55 U/L), gamma-glutamyl transferase (GGT) 356 U/L (8-61 U/L), total bilirubin 2.4 mg/dL (<1.2 mg/dL), and direct bilirubin 2 mg/dL (<0.3 mg/dL) (Table 1).

**Table 1:** Laboratory studies after hospitalization

Test	Normal	7 months	12 months	15 months
Bilirubin, total, mg/dL	<1.2	1.7	2.0	2.4
Alanine aminotransferase (ALT), U/L	7 - 55	274	147	133
Aspartate aminotransferase (AST), U/L	7 - 55	133	116	77
Alkaline phosphatase, IU/L	40 - 129	795	637	278
Gamma-glutamyl transferase (GGT), U/L	8 - 81			356

**Figure 1:**
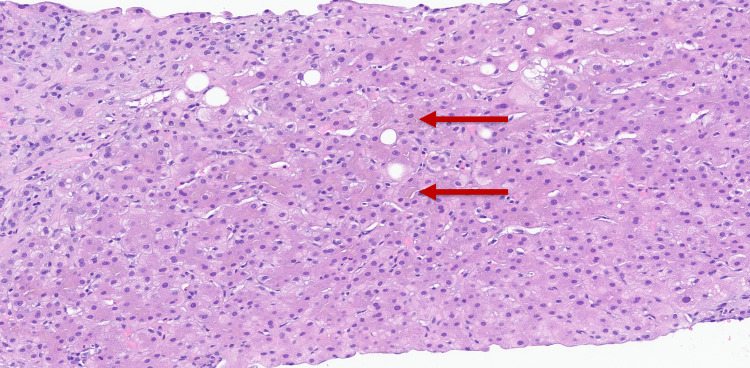
Liver biopsy, canalicular bile plugs are suggestive of chronic biliary disease

Autoimmune workup with anti-mitochondria and anti-smooth muscle antibody screens and hepatitis B and C screening was negative. Liver biopsy performed at the time of endoscopic retrograde cholangiopancreatography (ERCP) demonstrated bridging fibrosis in a biliary pattern with ductular proliferation and mixed inflammation and cholestasis, suggesting chronic biliary disease (Figure 1).

**Figure 2:**
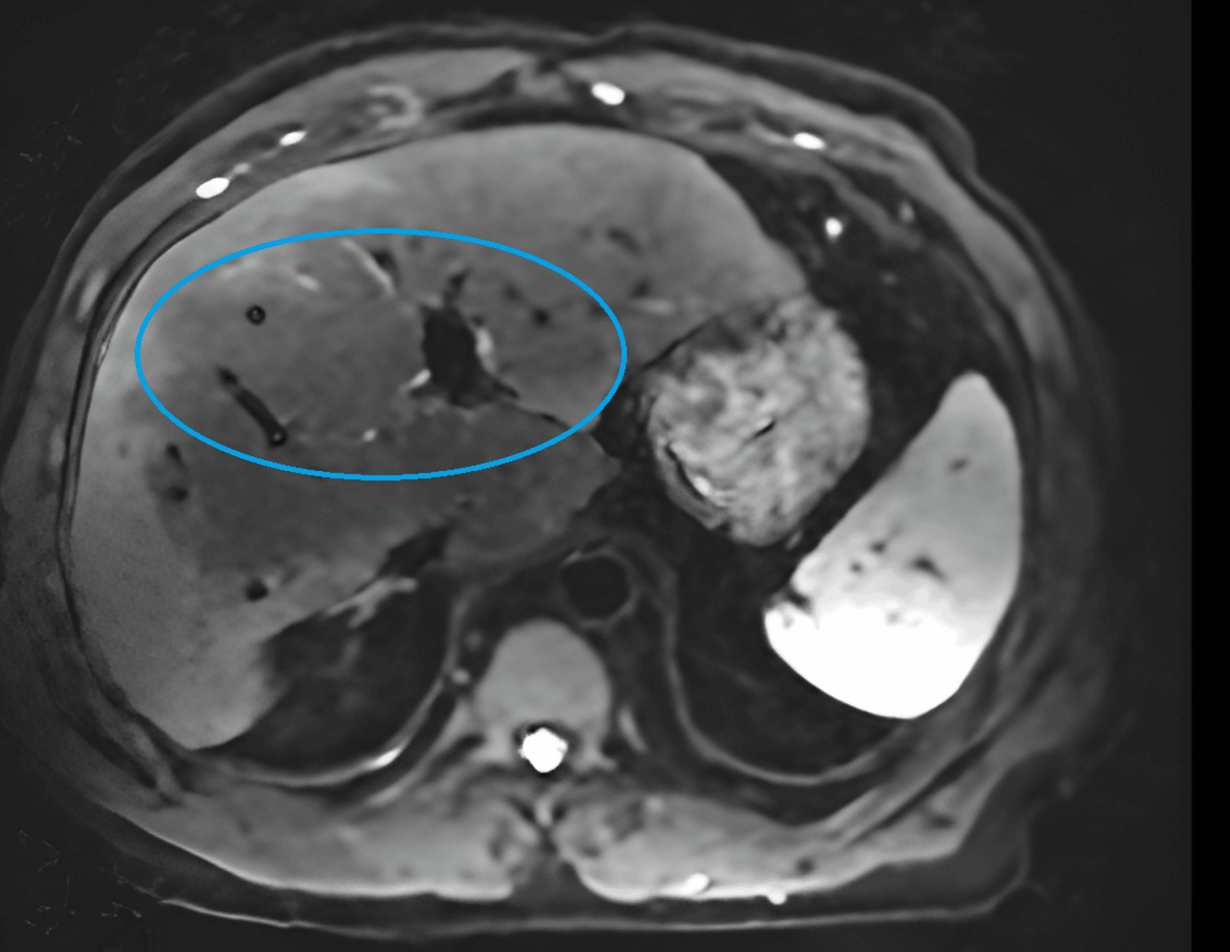
MRI of liver The MRI image demonstrates nodular hepatic contour suggestive of chronic parenchymal disease and peripheral geographic signal changes in the right hepatic lobe with capsular retraction are suggestive of confluent hepatic fibrosis.

He was diagnosed with autoimmune hepatitis and begun on 20 mg of prednisone daily and mycophenolate mofetil 1000 mg two times daily. Despite three months of therapy, his liver enzymes remained persistently elevated. At this point, he presented to our clinic for a second opinion. Magnetic resonance imaging (MRI) of the liver was performed and revealed stage 3-4 liver fibrosis and a nodular hepatic contour, suggesting chronic parenchymal disease (Figure 2).

**Figure 3:**
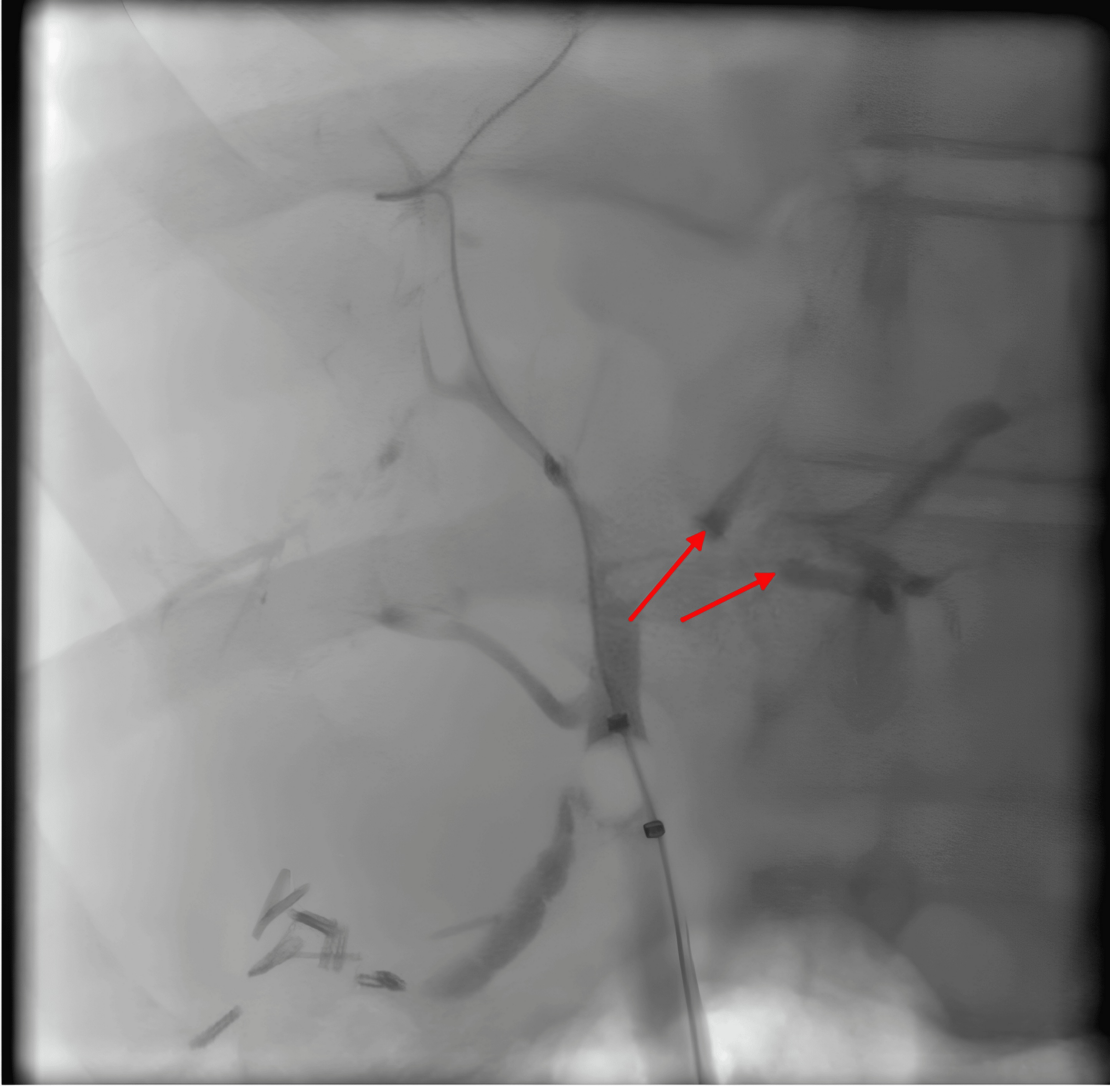
ERCP image Endoscopic retrograde cholangiopancreatography (ERCP) demonstrates diffuse findings consistent with secondary sclerosing cholangitis.

He was diagnosed with COVID-SSC. His steroid medication was tapered over 25 days. Given that his liver enzymes remained stable, the mycophenolate mofetil was then discontinued. The patient had two visits to the emergency room for increasing symptoms of pruritus, jaundice, and right upper quadrant pain. Hepatic ultrasound and computed tomography (CT) of the abdomen/pelvis did not reveal any acute pathologies and redemonstrated known liver cirrhosis. He began naltrexone and cholestyramine to treat his symptoms of cholestasis. He underwent a second ERCP for further evaluation, given persistently elevated liver enzymes and symptoms. This demonstrated diffuse changes of sclerosing cholangitis without any dominant strictures, and prior biliary sphincterotomy appeared open (Figure 3).

Choledocholithiasis at the left hepatic duct was removed. The patient remains on symptomatic treatment for pruritus and jaundice and is planned for regular imaging to screen for hepatobiliary malignancy, given his increased risk. He will be monitored for any indications of liver transplantation.

## Discussion

Though the pathogenesis of SSC-CIP has not been fully elucidated, a leading theory is ischemic cholangiopathy. While the hepatic parenchyma receives blood via the hepatic arteries and portal vein, the biliary epithelium is more susceptible to ischemic damage as it is supplied exclusively by the hepatic arteries. Factors common to ICU care that put patients at risk for biliary ischemia include hypotension and hemodynamic instability, hepatosplanchnic ischemia resulting from lung-protective mechanical ventilation, and microcirculatory disruption such as increased blood viscosity, RBC transfusions, and hypercoagulable states [4]. Changes in bile composition and biliary infections have also been posited. Patients with severe COVID-19 are exposed to many of these risk factors, suggesting a common genesis; however, COVID-SSC has also been identified in patients with moderate disease not requiring ICU cares [5]. It is well established that SARS-CoV-2 enters cells via the angiotensin converting enzyme 2 (ACE2) receptor. ACE2 expression levels are higher in bile duct cells and comparable to alveolar epithelial type II cells [6]. Studies utilizing human liver organoids demonstrated that cholangiocytes were susceptible to COVID-19 infection and supported efficient viral replication, suggesting a mechanism of direct viral damage [7]. Unique histologic features of COVID-SSC include degenerative cholangiocyte injury, cytoplasmic vacuolization, microvascular features of hepatic artery endothelial swelling, and regenerative changes, which are thought to also distinguish it from SSC-CIP [8].

The disease course of SSC typically begins with laboratory abnormalities followed by persistent cholestasis or recurrent bacterial cholangitis, and eventual progression of liver fibrosis and cirrhosis. In one study of 2,047 patients admitted for COVID-19, 12 were identified to develop cholestatic liver injury with a mean time of 118 days from COVID-19 diagnosis [9]. In a multicenter retrospective study of SSC, there was no significant difference in overall survival between patients with COVID-SSC and SSC-CIP, with 40% of COVID-SSC patients surviving transplant-free at the one-year mark [3].

## Conclusions

This case highlights an underrecognized entity known as SSC-CIP and its distinct variant, COVID-SSC, which most commonly occurs following COVID-19 infection requiring prolonged ICU care. Patients are more commonly male in the 4th to 5th decade of life and lack prior liver disease. This case highlights the importance of recognizing COVID-SSC as a potential etiology of asymptomatic alkaline-phosphatase predominant liver enzyme abnormalities upwards of one year after COVID-19 infection and exemplifies a more indolent disease course. Imaging should be considered for these patients as our patient was found to have cirrhosis at the time of diagnosis and will require regular screening for hepatobiliary malignancy given his increased risk.
